# Supplementary material for: MafB regulates NLRP3 inflammasome activation by sustaining p62 expression in macrophages
Source: Commun Biol. 2023 Oct 16;6:1047. doi: 10.1038/s42003-023-05426-5 (PMC10579372; doi:10.1038/s42003-023-05426-5)

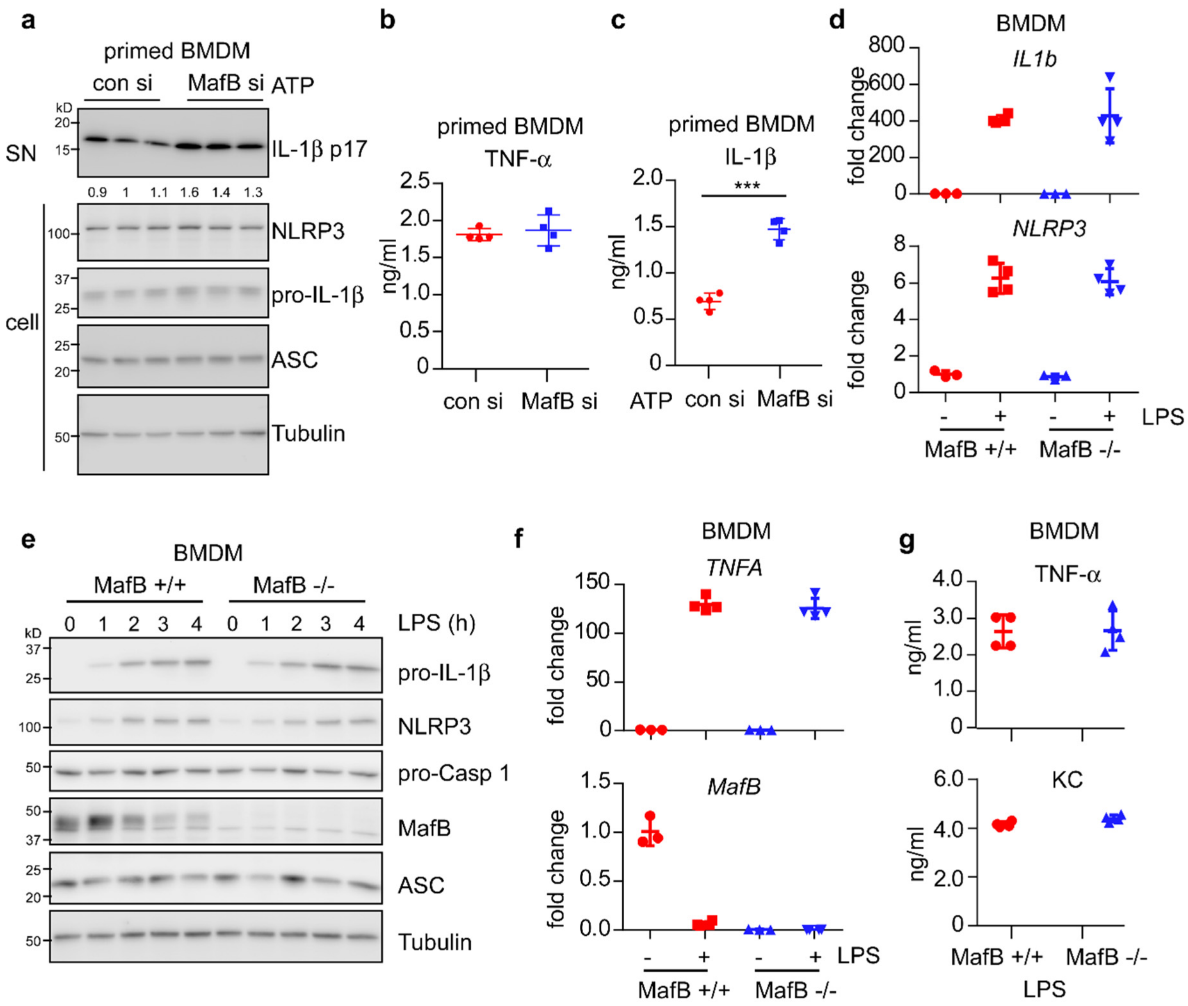

Supplementary Fig. 1 MafB does not participate in the LPS induced early pro-inflammatory response or the expression of NLRP3 and pro-IL-1β in BMDMs. (a-c) BMDMs transfected with control or MafB siRNAs were primed by LPS and stimulated with ATP. Levels of the indicated proteins in supernatants and cells were determined by Western blotting (a). Supernatant TNF-α (b) and IL-1β (c) levels were determined by ELISA. (d-e) MafB +/+ and MafB -/- BMDMs were treated with or without LPS. mRNA levels of the indicated genes were determined by real-time PCR (d). Levels of the indicated proteins in the cells were determined by Western blotting (e). (f-g) MafB +/+ and MafB -/- BMDMs were treated with or without LPS. mRNA levels of the indicated genes were determined by real-time PCR (f). Supernatant TNF-α and KC (g) levels were determined by ELISA. Vertically stacked bands originate either from a single membrane or from a replicate membrane, with the same sample loadings. Mean±SD; *** $p<0.001$. Representative of two independent experiments.

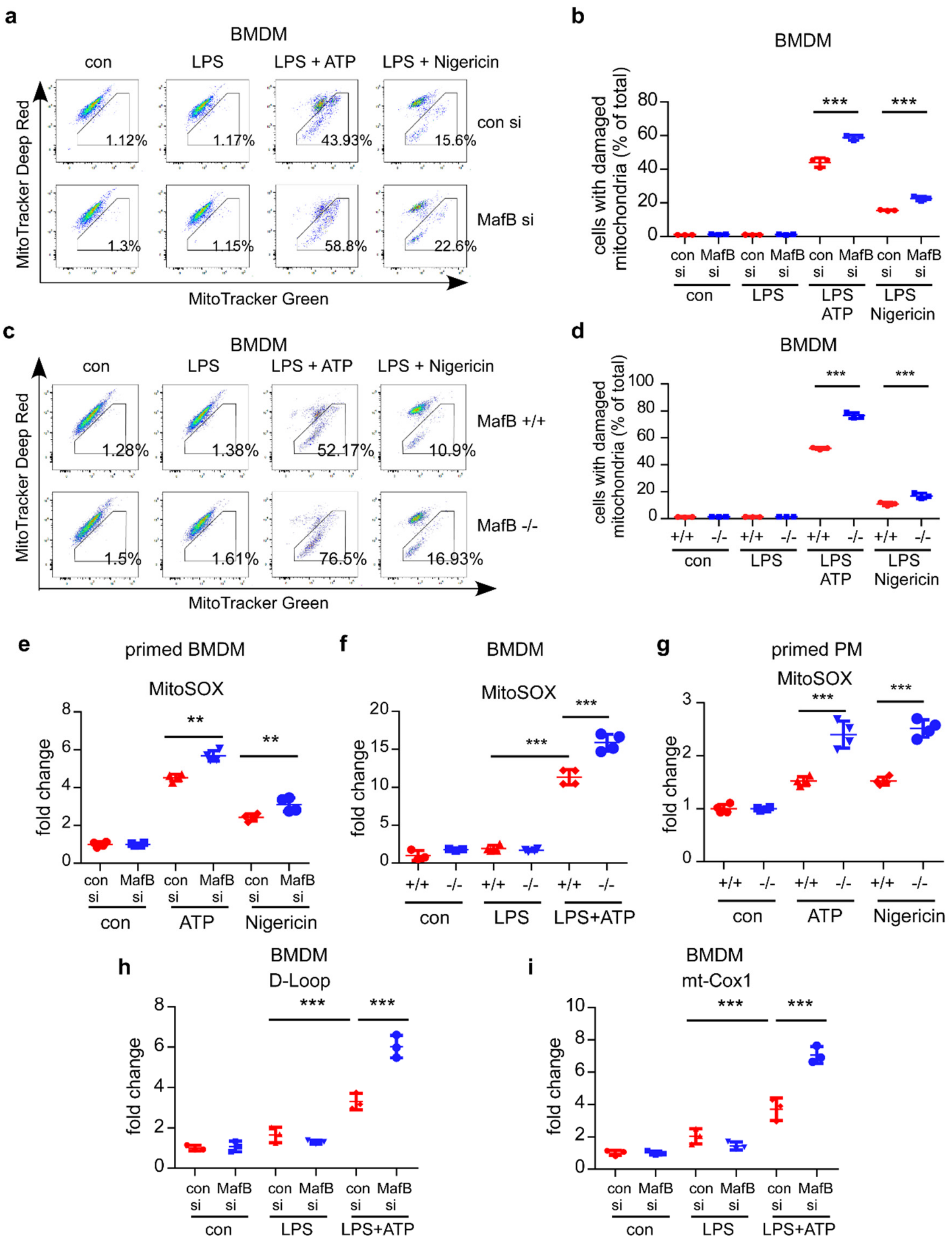

Supplementary Fig. 2 MafB restricts the NLRP3 agonists induced mitochondrial damage in BMDMs and PMs. (a-b) BMDMs were transfected with control siRNAs or MafB siRNAs. 48h later, cells were treated with or without LPS, stimulated with ATP or Nigericin, and stained with MitoTracker Deep Red and MitoTracker Green. Cells were collected, and flow cytometric analysis was performed to determine the mitochondria damage. (c-d) MafB +/+ and MafB -/- BMDMs

were treated like those in a and mitochondrial damage was evaluated by flow cytometry. (e-g) Primed BMDMs transfected with control or MafB siRNAs (e) and primed MafB +/+ and MafB -/- BMDMs (f) and PMs (g) were incubated with MitoSOX for 15 min, followed by stimulation with ATP or Nigericin for 30-60 min. mtROS was determined by fluorescence microplate reader with Ex530/Em590 nm. (h-g) BMDMs transfected with control siRNAs or MafB siRNAs were primed with LPS, followed by stimulation with ATP. Cytoplasmic DNAs were prepared and levels of mtDNA were determined by real-time PCR assay. Mean±SD ** $p<0.01$, *** $p<0.001$. Representative of two independent experiments.

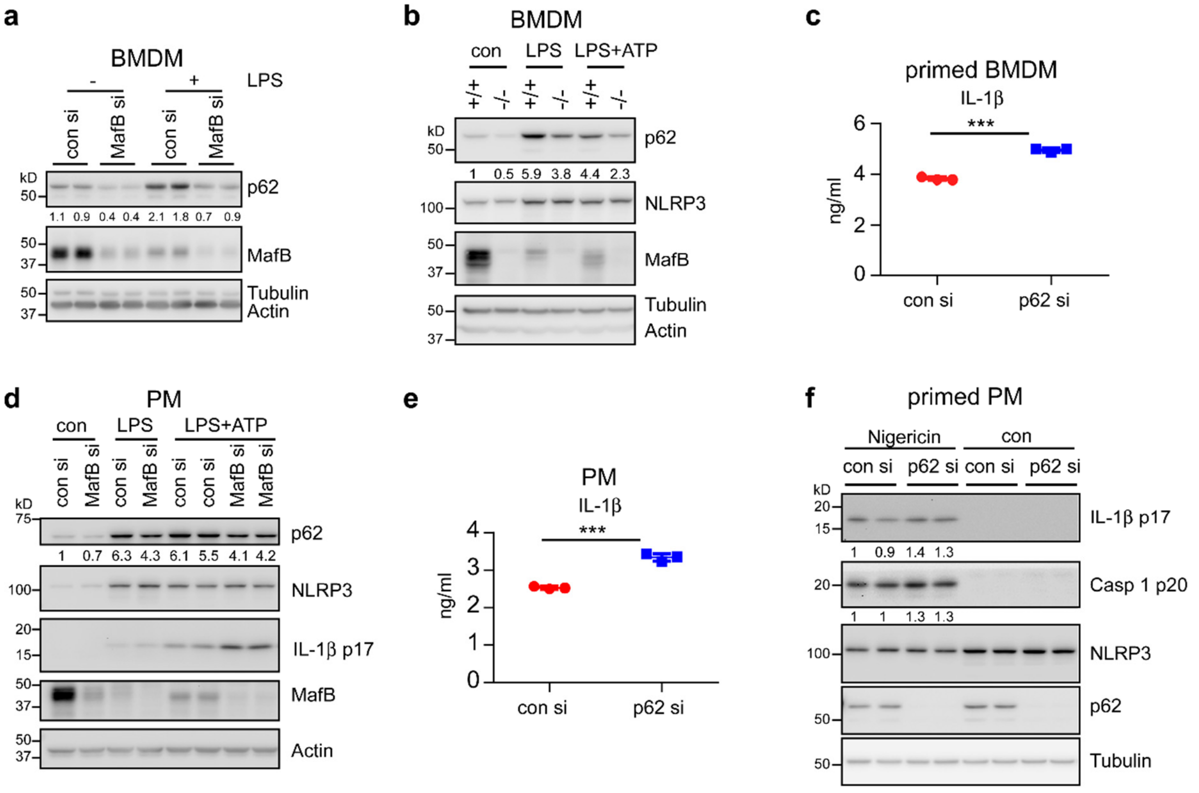

Supplementary Fig. 3 MafB sustains p62 expression in in BMDMs and PMs. (a) BMDMs transfected with control siRNAs or MafB siRNAs were treated with or without LPS. Levels of the indicated proteins were determined by Western blotting. (b) MafB +/+ and MafB -/- BMDMs were treated with or without LPS, followed by stimulation with ATP. (c) BMDMs transfected with control siRNAs or p62 siRNAs were primed with LPS, followed by stimulation with ATP. Supernatant IL-1β was determined by ELISA. (d) PMs transfected with control siRNAs or MafB siRNAs were treated with or without LPS, followed by stimulation with ATP. Western blotting was performed. (e-f) PMs transfected with control siRNAs or p62 siRNAs were primed with LPS, followed by stimulation with Nigericin. Supernatant IL-1β was determined by ELISA (e). Levels of the indicated proteins were determined by Western blotting (f). Mean±SD; *** $p<0.001$. Representative of two independent experiments.

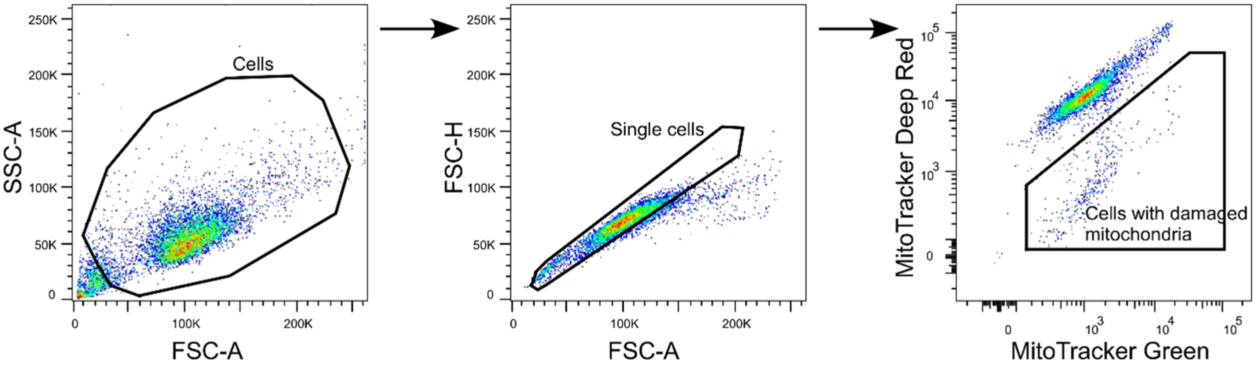

Supplementary Fig. 4 Gating strategy for flow cytometric analysis of mitochondrial damage in macrophages.

Supplementary Figure 5. Uncropped blots

Representative uncropped images, alongside respective membrane counterparts, show the accuracy of the size marker labeling in the figure.

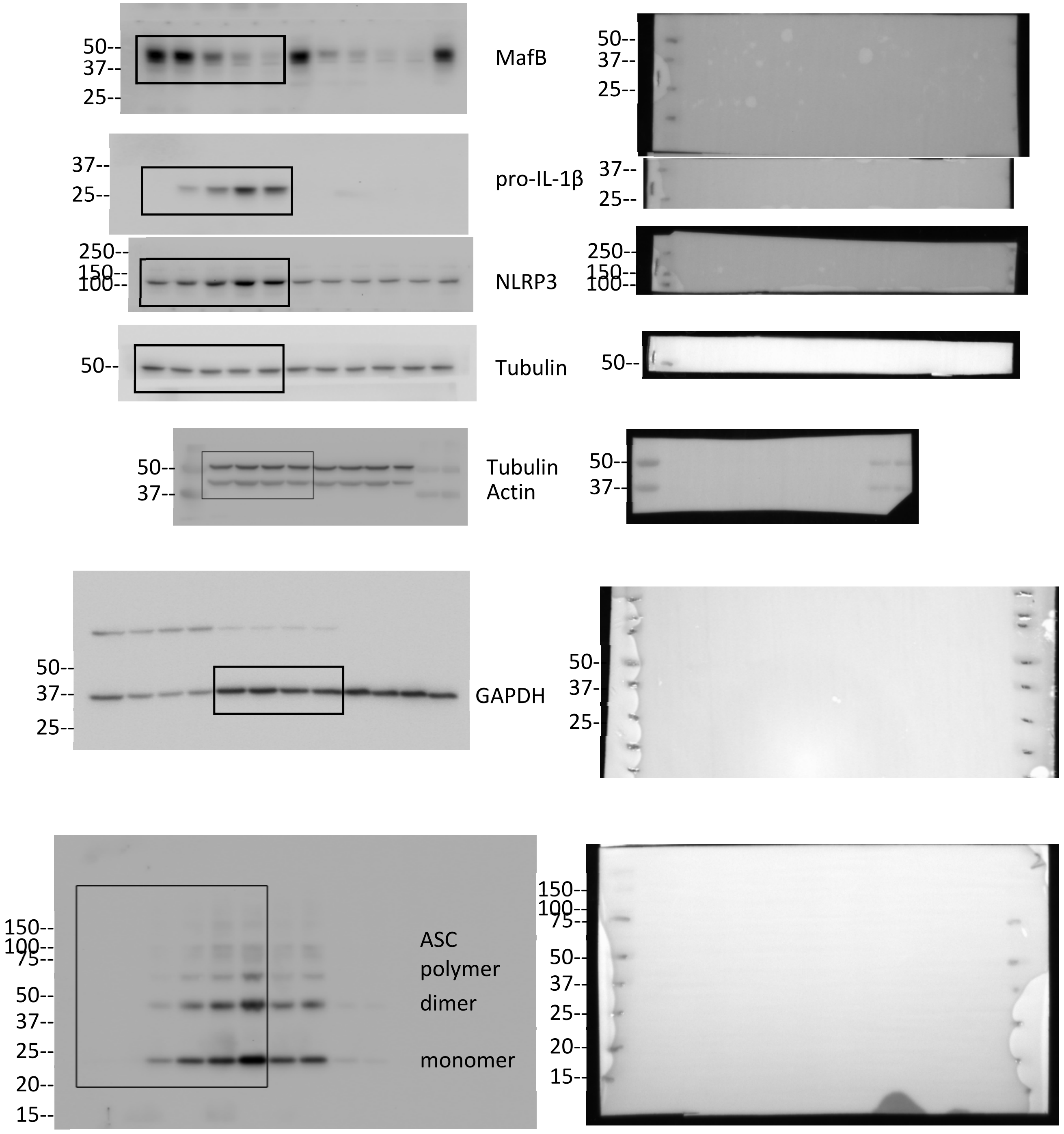

Supplementary Figure 5. Uncropped blots

Representative uncropped images, alongside respective membrane counterparts, show the accuracy of the size marker labeling in the figure.

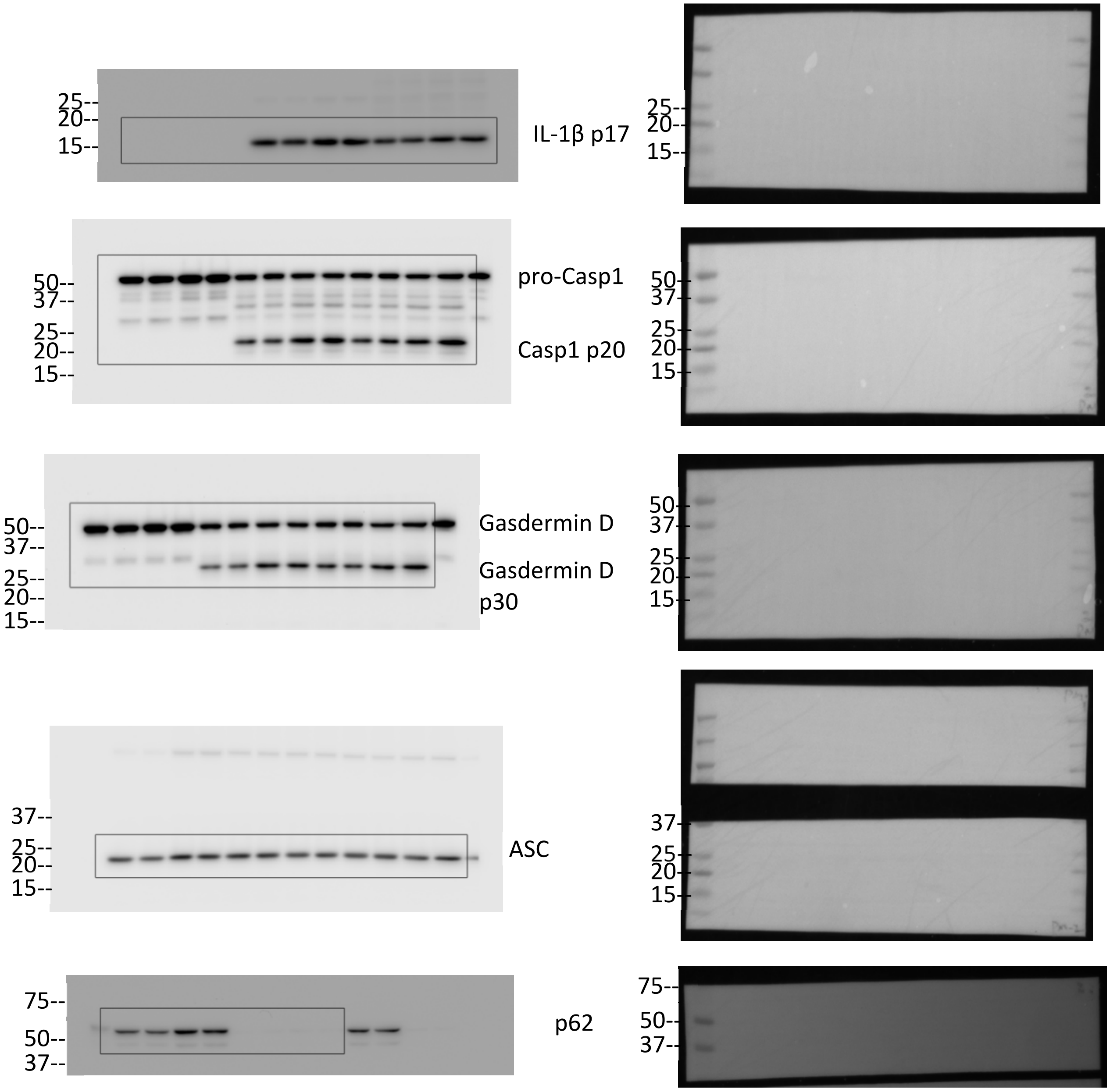

Supplementary Figure 5. Uncropped blots

Figure 1

1a

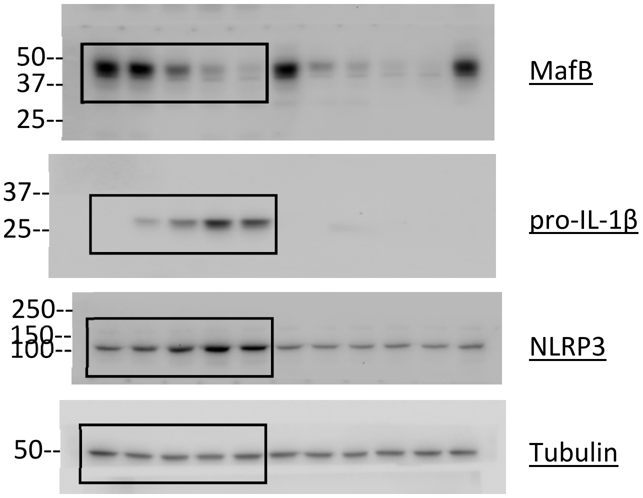

1b

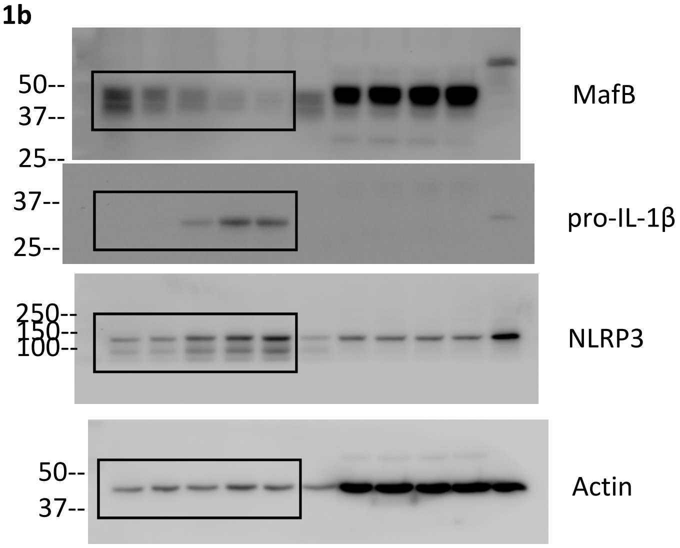

1c

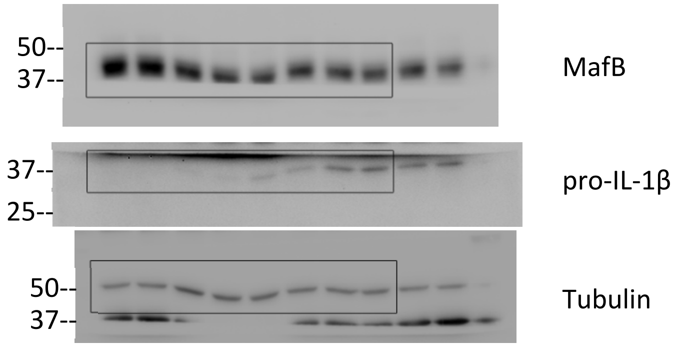

1f

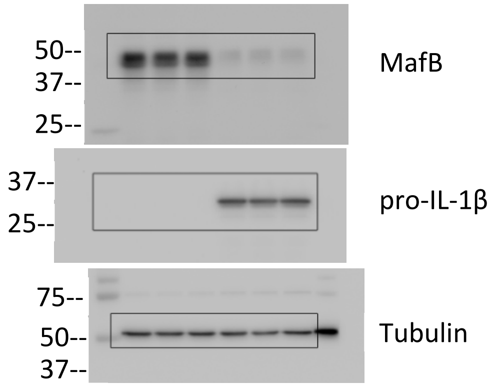

1g

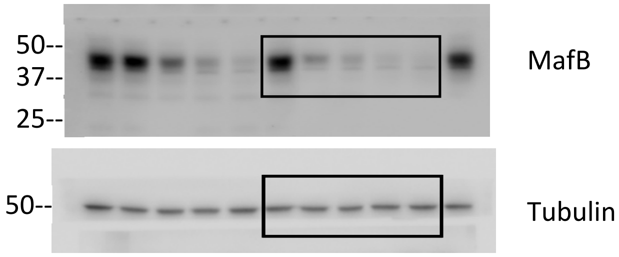

1h

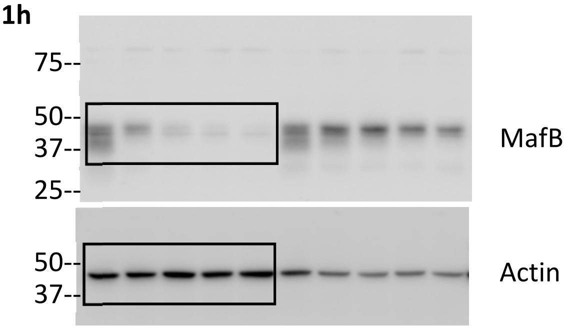

Figure 1

1i

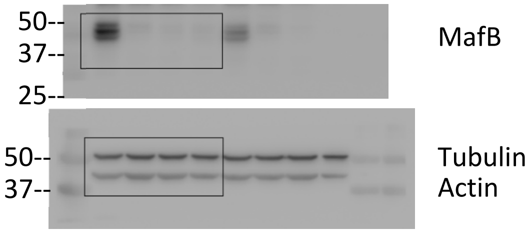

1j

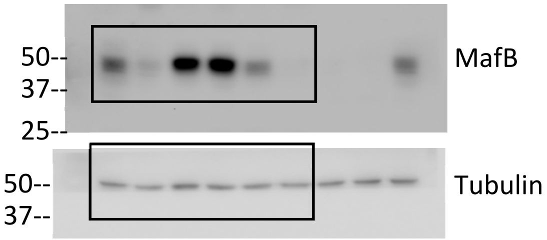

1l

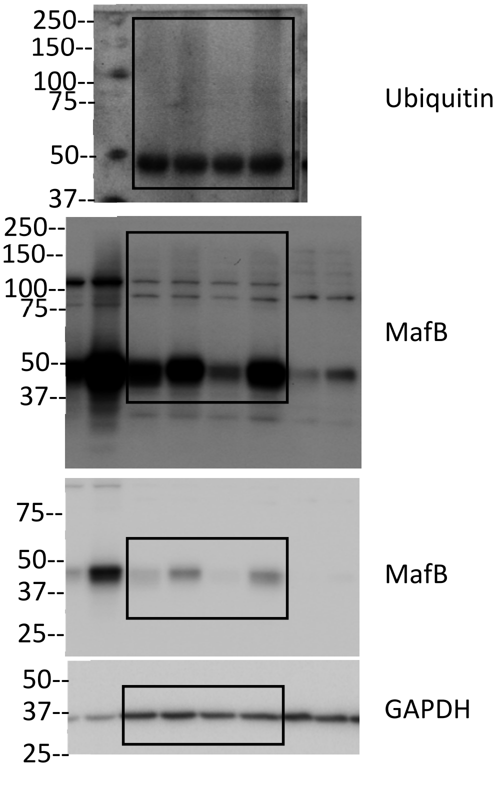

1k

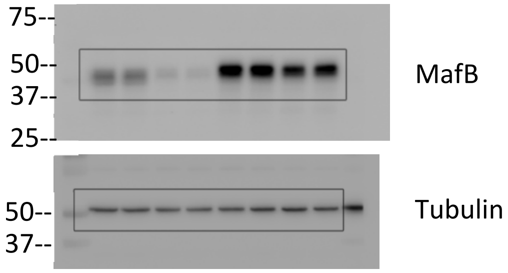

Figure 2

2a

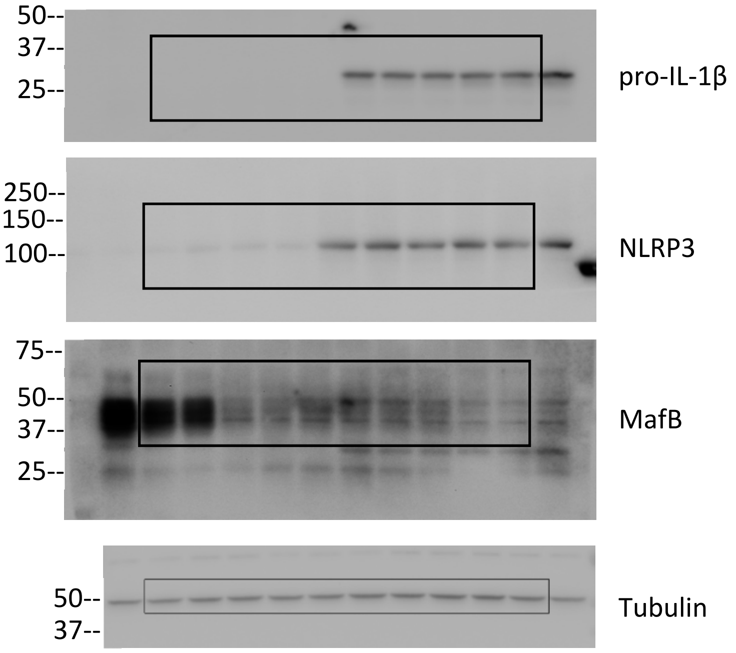

2f

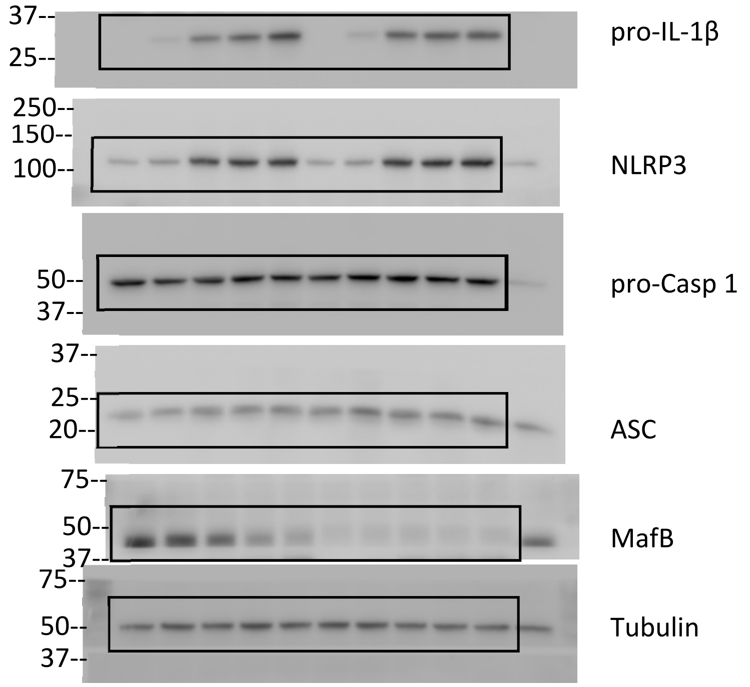

Figure 3

3d

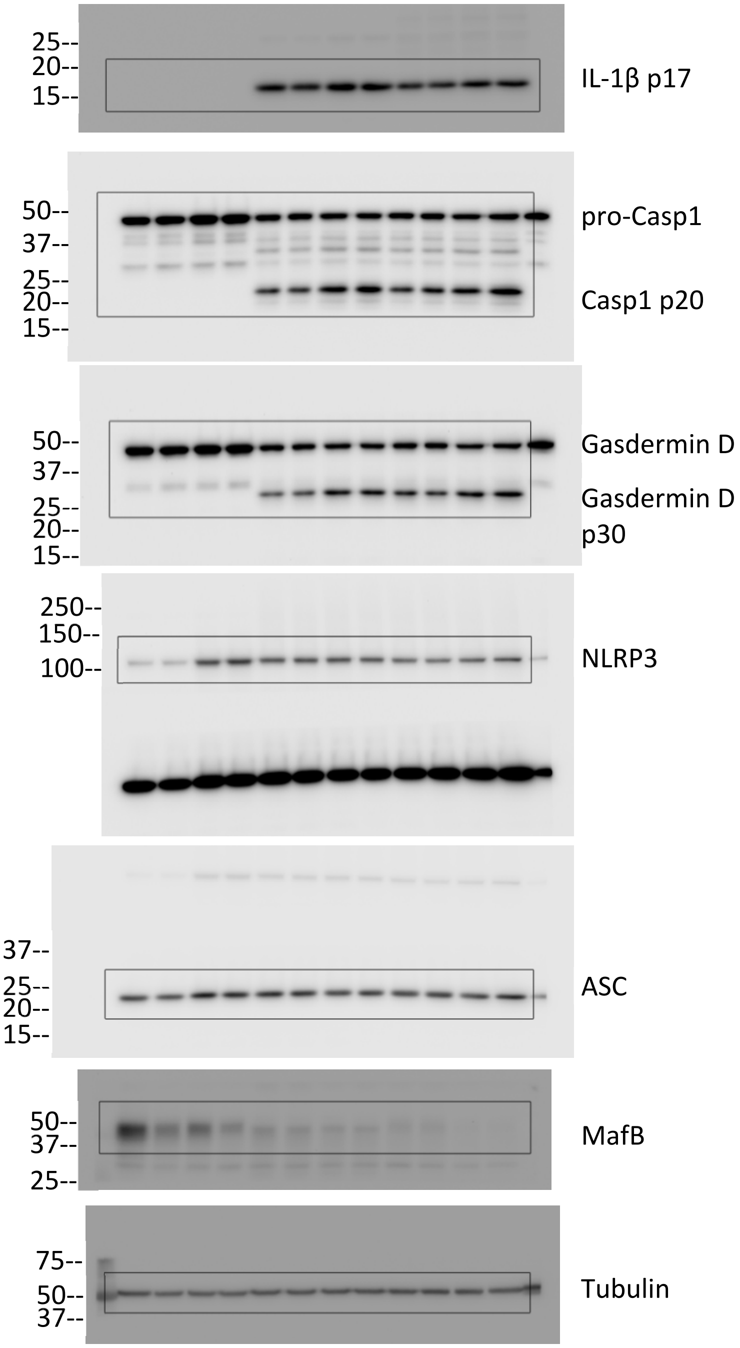
25--
20--
15--
IL-1β p17
50--
37--
25--
20--
15--
pro-Casp1
Casp1 p20
50--
37--
25--
20--
15--
Gasdermin D
Gasdermin D p30
250--
150--
100--
NLRP3
37--
25--
20--
15--
ASC
50--
37--
25--
MafB
75--
50--
37--
Tubulin

3h

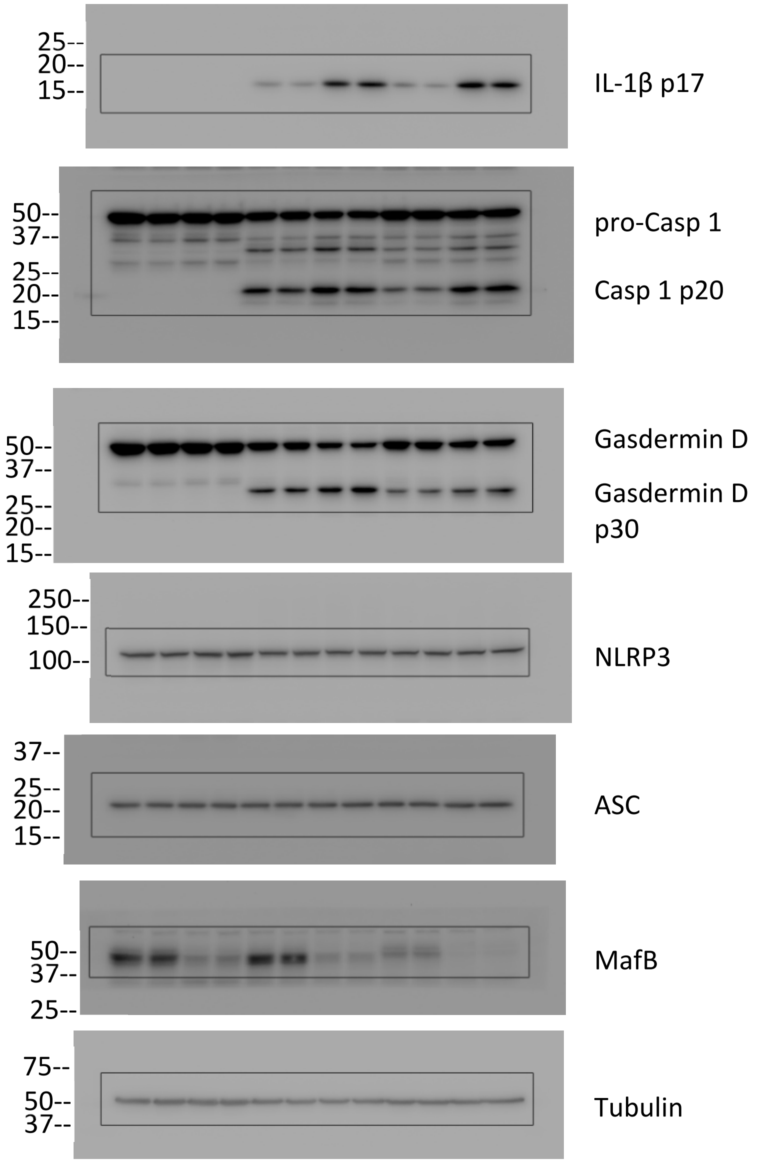

25--
20--
15--
IL-1β p17
50--
37--
25--
20--
15--
pro-Casp 1
Casp 1 p20
50--
37--
25--
20--
15--
Gasdermin D
Gasdermin D p30
250--
150--
100--
NLRP3
37--
25--
20--
15--
ASC
50--
37--
25--
MafB
75--
50--
37--
Tubulin

Figure 4

4b

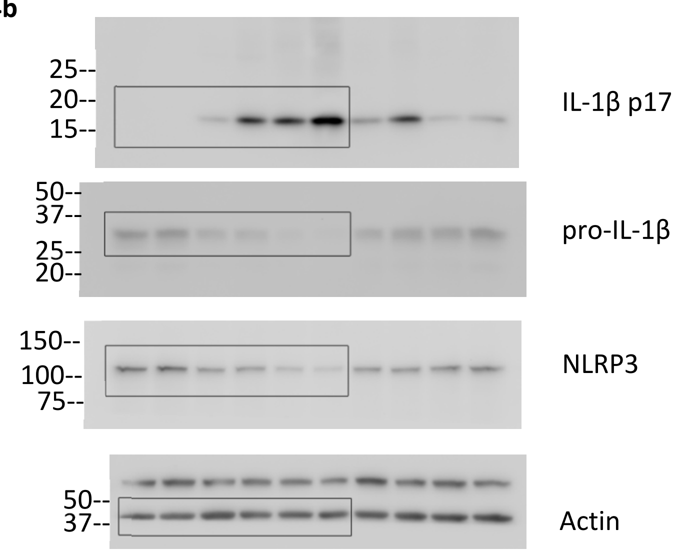

4d

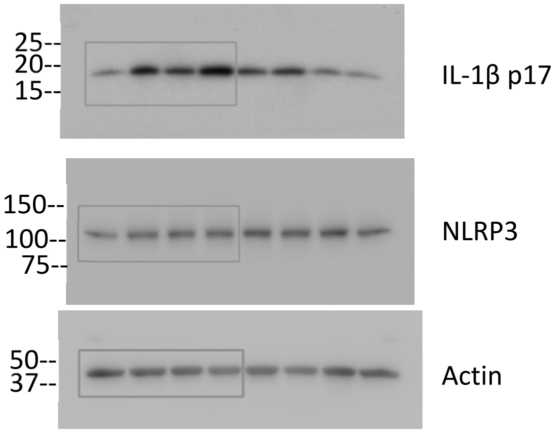

4e

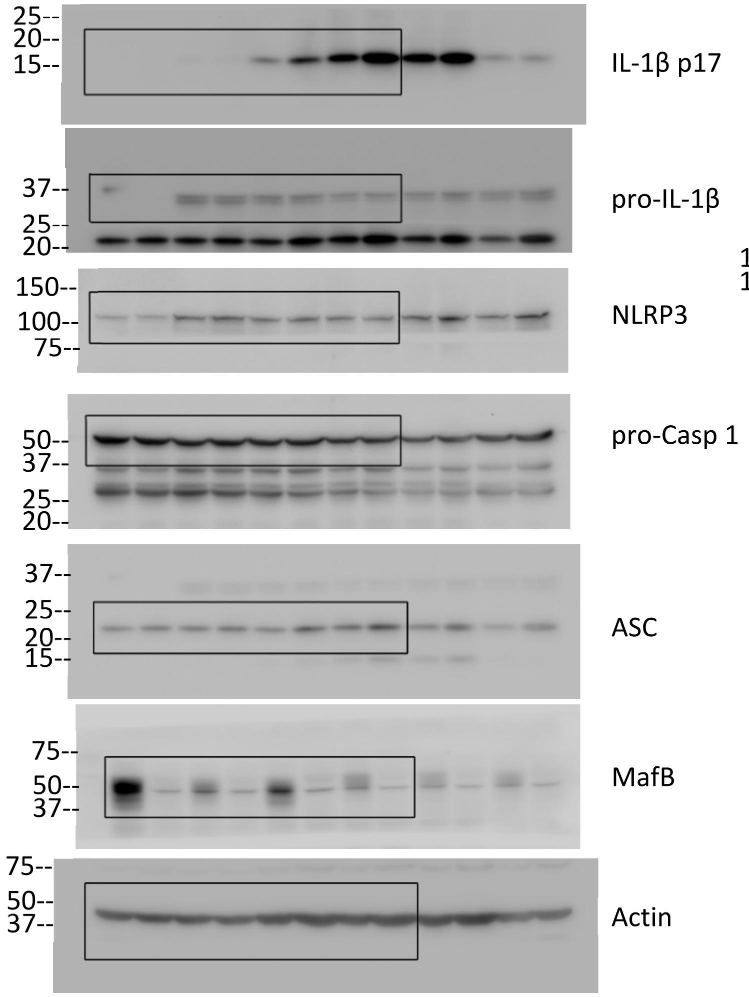

4f

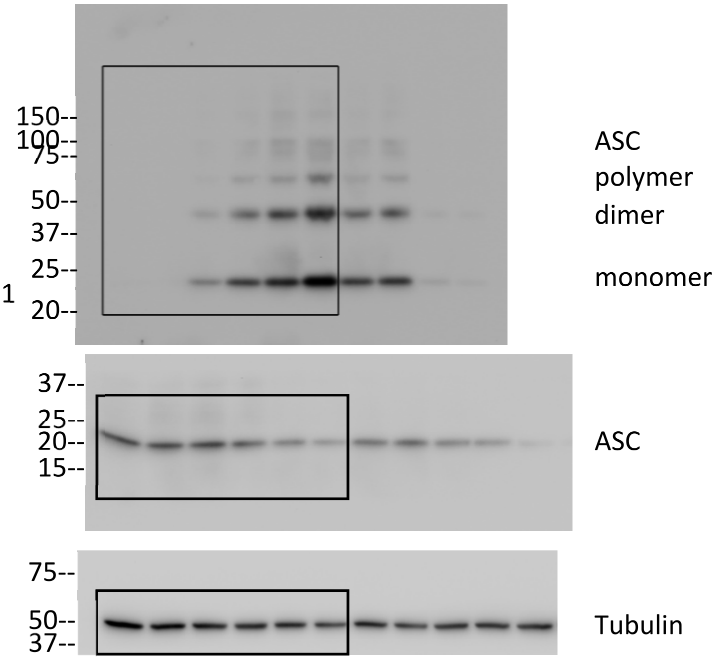

Figure 4

4i

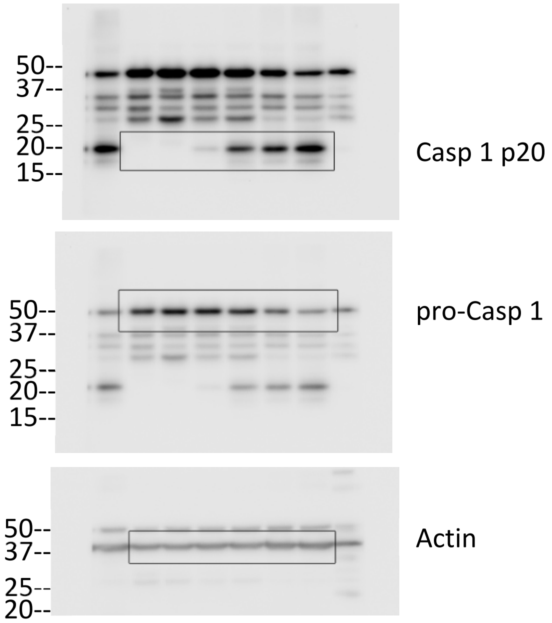

4j

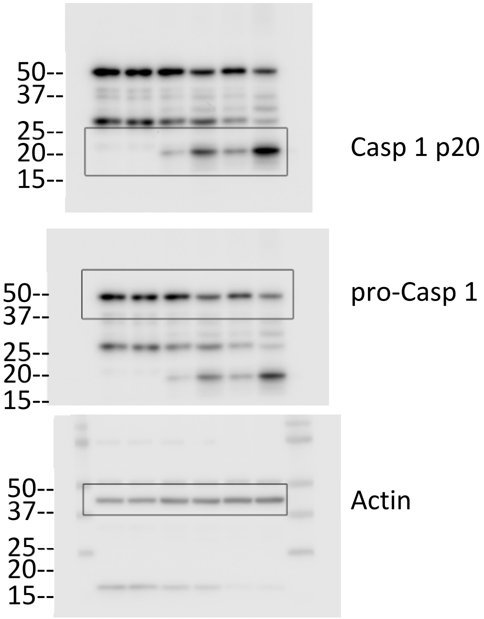

4k

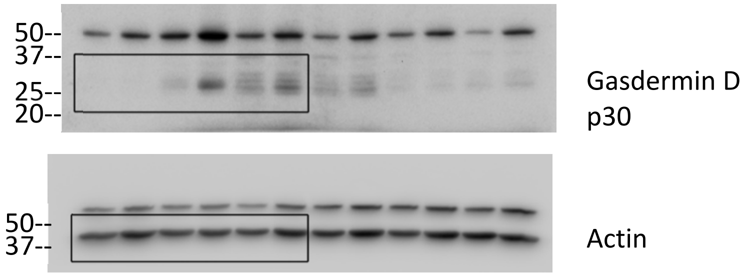

Figure 5

5c

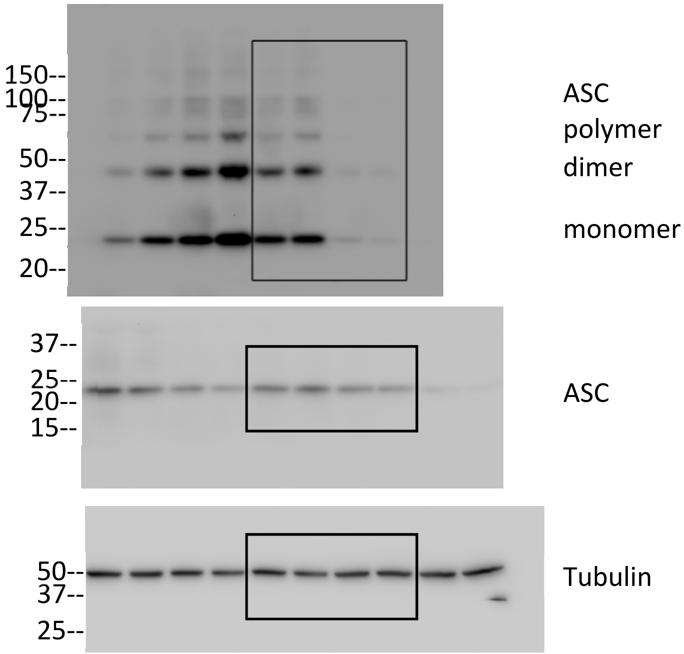

5b

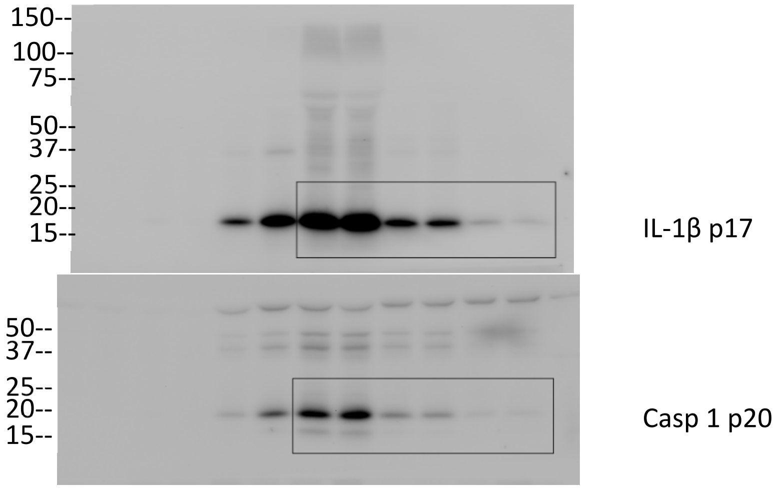

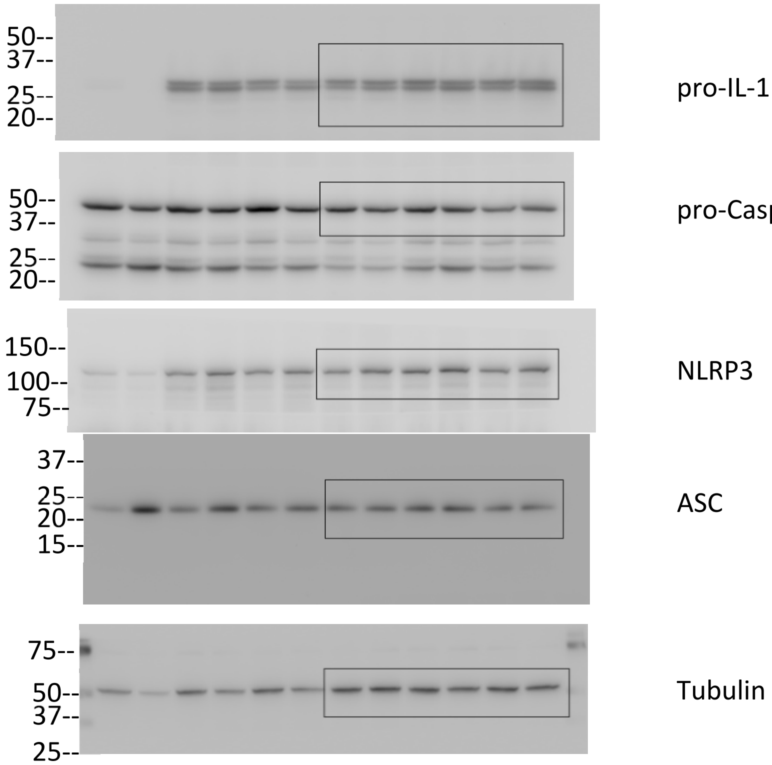

5e

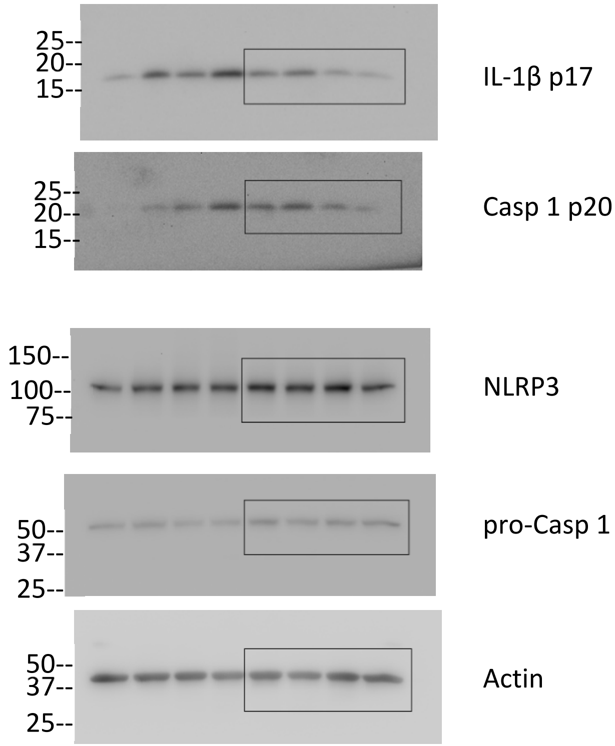

Figure 6

6g

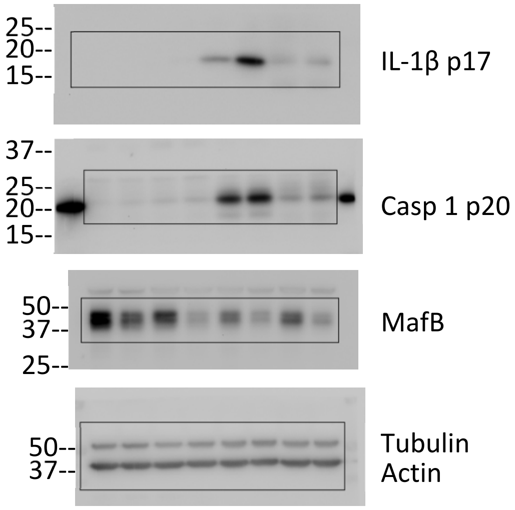
25--
20--
15--
IL-1β p17
37--
25--
20--
15--
Casp 1 p20
50--
37--
25--
MafB
50--
37--
Tubulin
Actin

Figure 7

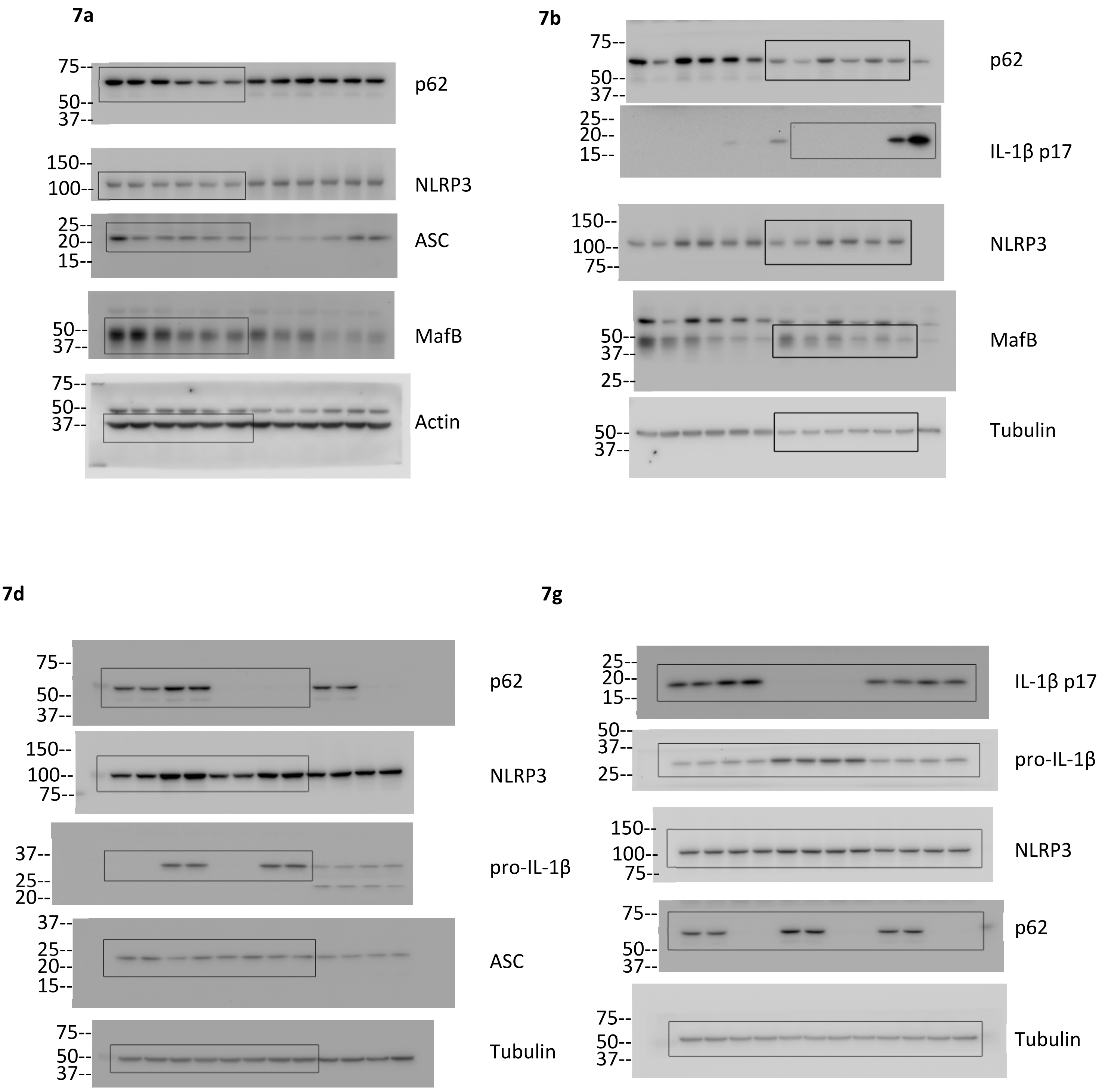
7a
75--
50--
37--
p62
150--
100--
NLRP3
25--
20--
15--
ASC
50--
37--
MafB
75--
50--
37--
Actin
7b
75--
50--
37--
p62
25--
20--
15--
IL-1β p17
150--
100--
75--
NLRP3
50--
37--
25--
MafB
50--
37--
Tubulin
7d
75--
50--
37--
p62
150--
100--
75--
NLRP3
37--
25--
20--
pro-IL-1β
37--
25--
20--
15--
ASC
75--
50--
37--
Tubulin
7g
25--
20--
15--
IL-1β p17
50--
37--
25--
pro-IL-1β
150--
100--
75--
NLRP3
75--
50--
37--
p62
75--
50--
37--
Tubulin

Figure 8

8a

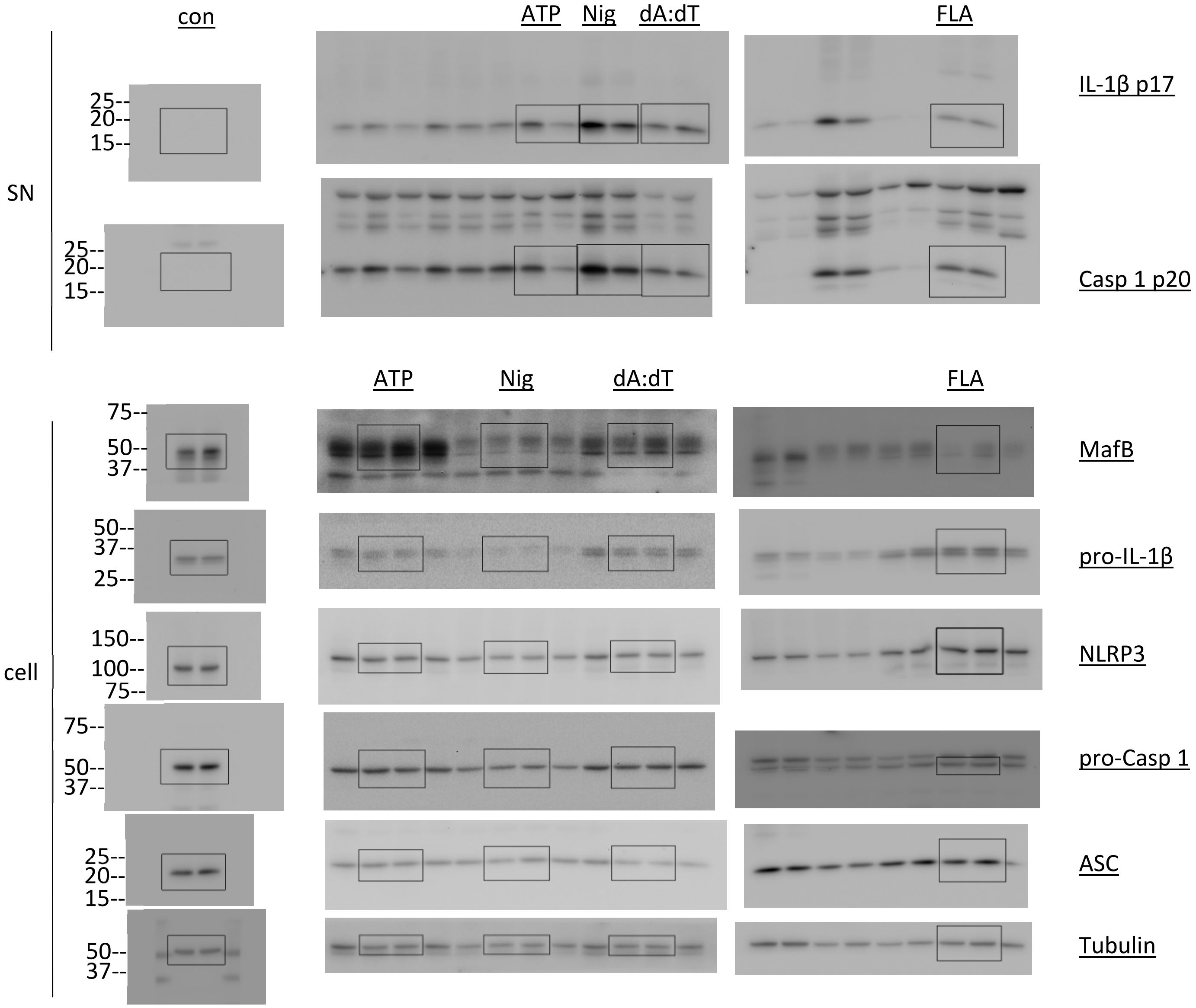
con
ATP Nig dA:dT
FLA
25--
20--
15--
IL-1β p17
SN
25--
20--
15--
Casp 1 p20
ATP Nig dA:dT
FLA
75--
50--
37--
MafB
50--
37--
25--
pro-IL-1β
150--
100--
75--
NLRP3
cell
75--
50--
37--
pro-Casp 1
25--
20--
15--
ASC
50--
37--
Tubulin

Figure 8

8d

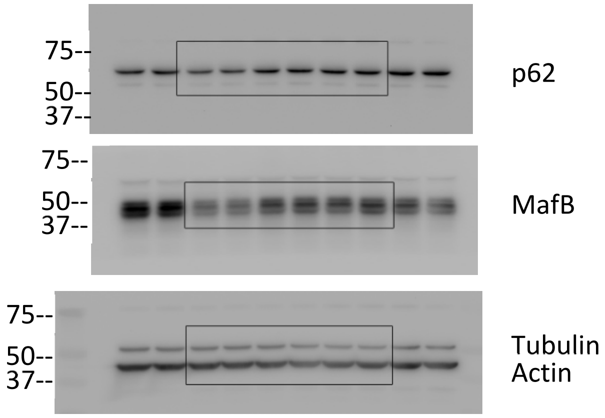

8e

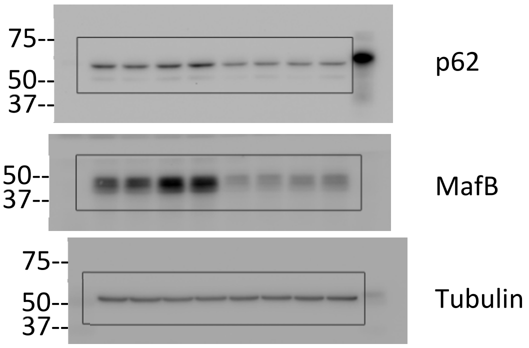

8f

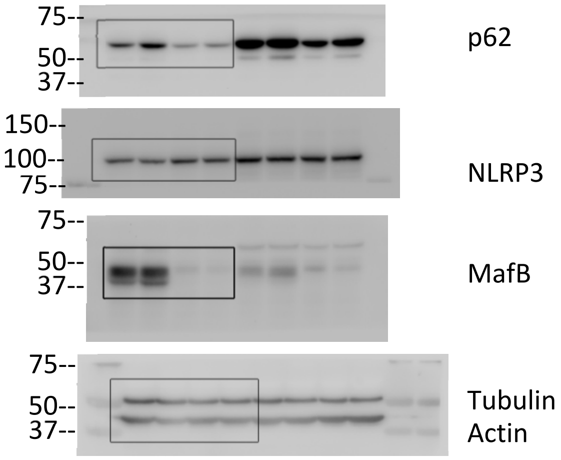

Figure 9

9c

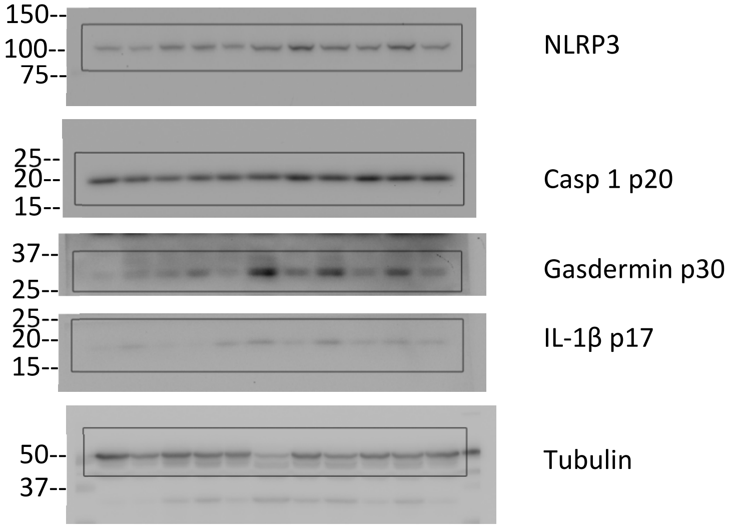
150--
100--
75--
NLRP3
25--
20--
15--
Casp 1 p20
37--
25--
Gasdermin p30
25--
20--
15--
IL-1β p17
50--
37--
Tubulin

Supplementary Figure 1

1a

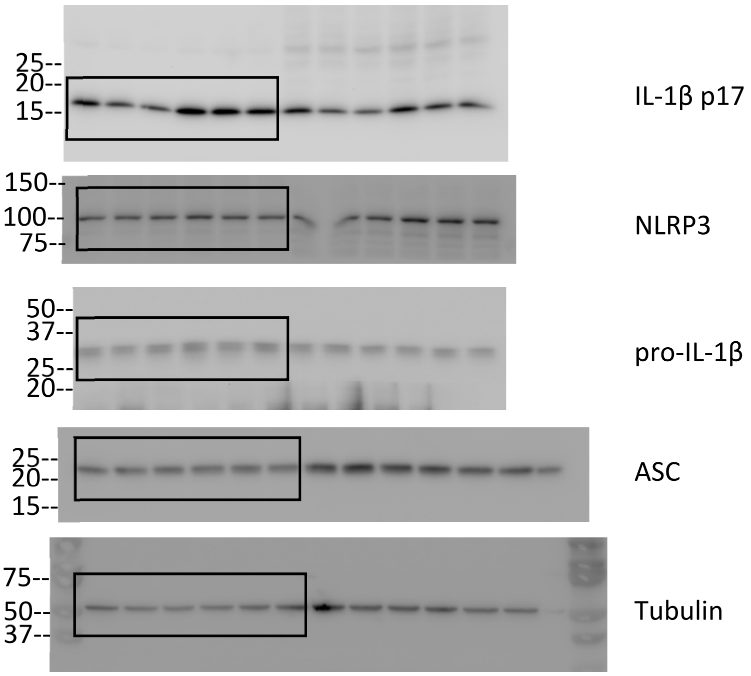

1e

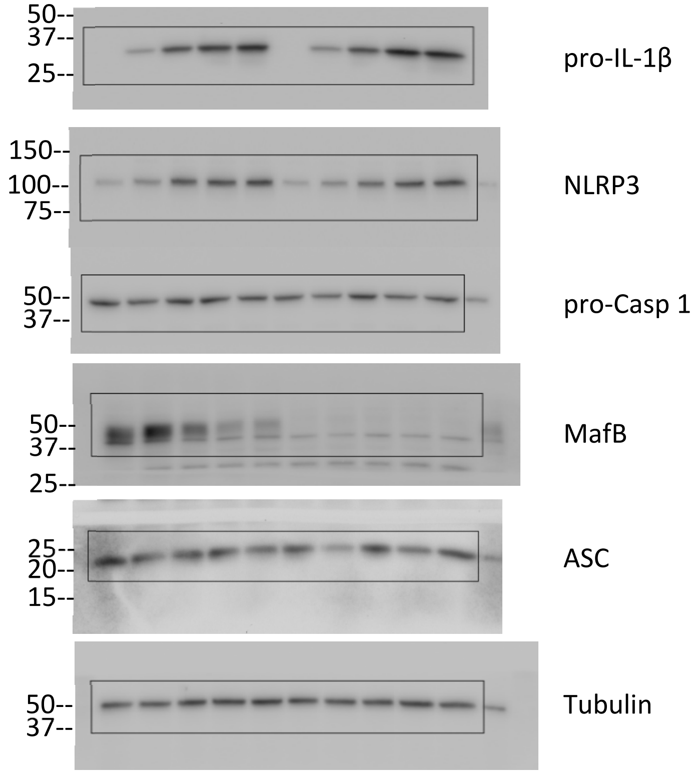

Supplementary Figure 3

3a

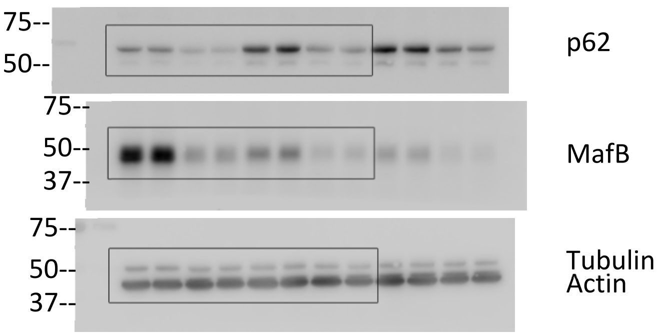

3b

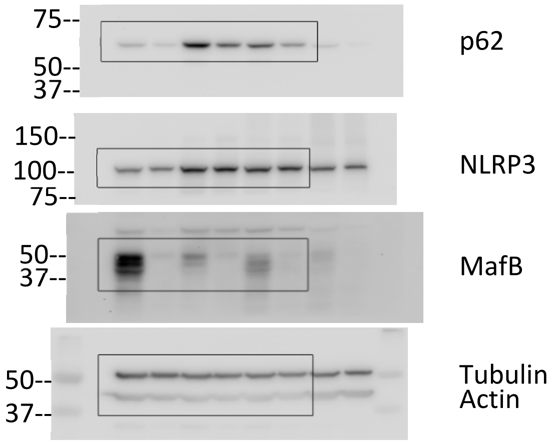

3d

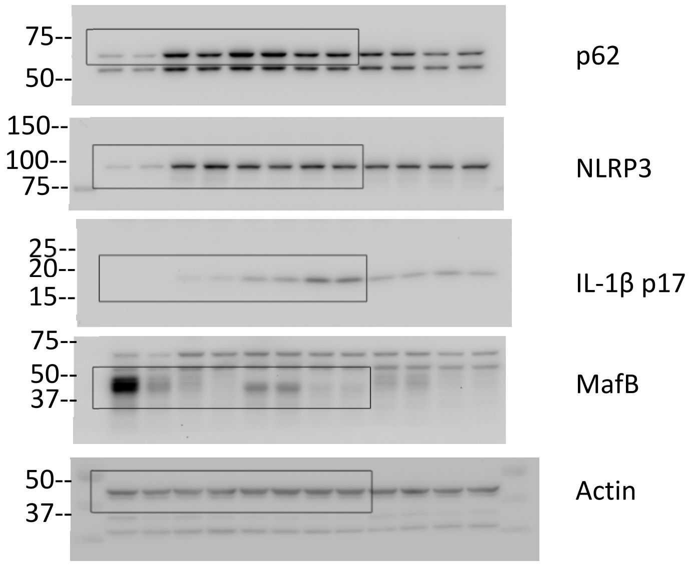

3f

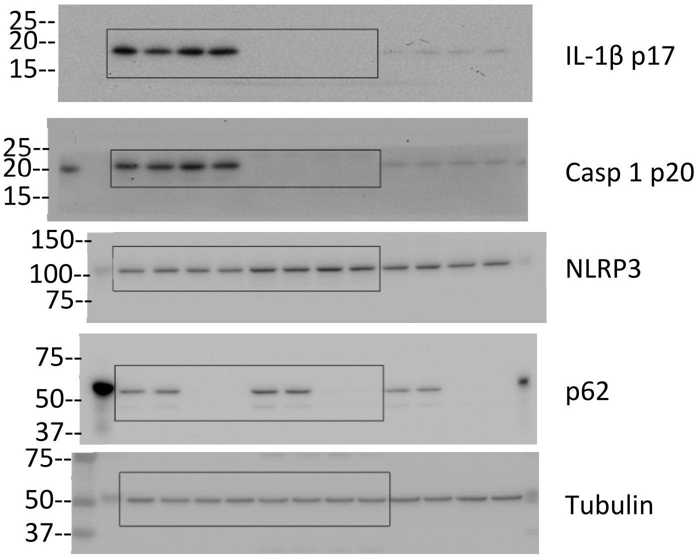

Supplement: Supplementary file 2 — Supplementary Information [file 42003_2023_5426_MOESM2_ESM.pdf]
