# Supplementary material for: MafB regulates NLRP3 inflammasome activation by sustaining p62 expression in macrophages
Source: Commun Biol. 2023 Oct 16;6:1047. doi: 10.1038/s42003-023-05426-5 (PMC10579372; doi:10.1038/s42003-023-05426-5)
Supplement: Supplementary file 5 — Reporting Summary [file 42003_2023_5426_MOESM5_ESM.pdf]

nature portfolio

Corresponding author(s): Gang Liu

Last updated by author(s): Sep 25, 2023

Reporting Summary

Nature Portfolio wishes to improve the reproducibility of the work that we publish. This form provides structure for consistency and transparency in reporting. For further information on Nature Portfolio policies, see our Editorial Policies and the Editorial Policy Checklist.

Statistics

For all statistical analyses, confirm that the following items are present in the figure legend, table legend, main text, or Methods section.

n/a	Confirmed	
☐	☒	The exact sample size (*n*) for each experimental group/condition, given as a discrete number and unit of measurement
☐	☒	A statement on whether measurements were taken from distinct samples or whether the same sample was measured repeatedly
☐	☒	The statistical test(s) used AND whether they are one- or two-sided<br>*Only common tests should be described solely by name; describe more complex techniques in the Methods section.*
☐	☒	A description of all covariates tested
☐	☒	A description of any assumptions or corrections, such as tests of normality and adjustment for multiple comparisons
☐	☒	A full description of the statistical parameters including central tendency (e.g. means) or other basic estimates (e.g. regression coefficient) AND variation (e.g. standard deviation) or associated estimates of uncertainty (e.g. confidence intervals)
☒	☐	For null hypothesis testing, the test statistic (e.g. *F*, *t*, *r*) with confidence intervals, effect sizes, degrees of freedom and *P* value noted<br>*Give P values as exact values whenever suitable.*
☒	☐	For Bayesian analysis, information on the choice of priors and Markov chain Monte Carlo settings
☒	☐	For hierarchical and complex designs, identification of the appropriate level for tests and full reporting of outcomes
☒	☐	Estimates of effect sizes (e.g. Cohen's *d*, Pearson's *r*), indicating how they were calculated

Our web collection on statistics for biologists contains articles on many of the points above.

Software and code

Policy information about availability of computer code

Data collection	Bio-Rad CFX384 qPCR Instrument, Bio-Rad ChemiDoc Imaging system, BD FACSymphon A3, BioTek Synergy Mx Microplate Reader, Olympus ix73 microscope
Data analysis	GraphPad Prism 8, Microsoft 365 (Excel), FlowJo v10, ImageJ

For manuscripts utilizing custom algorithms or software that are central to the research but not yet described in published literature, software must be made available to editors and reviewers. We strongly encourage code deposition in a community repository (e.g. GitHub). See the Nature Portfolio guidelines for submitting code & software for further information.

Data

Policy information about availability of data

All manuscripts must include a data availability statement. This statement should provide the following information, where applicable:

- Accession codes, unique identifiers, or web links for publicly available datasets
- A description of any restrictions on data availability
- For clinical datasets or third party data, please ensure that the statement adheres to our policy

All data of this study are available upon request from the corresponding author.

Research involving human participants, their data, or biological material

Policy information about studies with human participants or human data. See also policy information about sex, gender (identity/presentation), and sexual orientation and race, ethnicity and racism.

Reporting on sex and gender	Not applicable
Reporting on race, ethnicity, or other socially relevant groupings	Not applicable
Population characteristics	Not applicable
Recruitment	Not applicable
Ethics oversight	Not applicable

Note that full information on the approval of the study protocol must also be provided in the manuscript.

Field-specific reporting

Please select the one below that is the best fit for your research. If you are not sure, read the appropriate sections before making your selection.

☒ Life sciences ☐ Behavioural & social sciences ☐ Ecological, evolutionary & environmental sciences

For a reference copy of the document with all sections, see nature.com/documents/nr-reporting-summary-flat.pdf

Life sciences study design

All studies must disclose on these points even when the disclosure is negative.

Sample size	Samples size were determined based on our previous studies on similar experiments.
Data exclusions	No data were excluded from analysis.
Replication	Experiments were repeated for three times, unless otherwise specified.
Randomization	No randomization was used.
Blinding	Investigators were blinded during the acquisition and quantification of fluorescence microscopy images.

Reporting for specific materials, systems and methods

We require information from authors about some types of materials, experimental systems and methods used in many studies. Here, indicate whether each material, system or method listed is relevant to your study. If you are not sure if a list item applies to your research, read the appropriate section before selecting a response.

Materials & experimental systems

n/a	Involved in the study	
☐	☒	Antibodies
☐	☒	Eukaryotic cell lines
☒	☐	Palaeontology and archaeology
☐	☒	Animals and other organisms
☒	☐	Clinical data
☒	☐	Dual use research of concern
☒	☐	Plants

Methods

n/a	Involved in the study	
☒	☐	ChIP-seq
☐	☒	Flow cytometry
☒	☐	MRI-based neuroimaging

Antibodies

Antibodies used	Mouse anti-α-tubulin antibody (catlog T5168) and mouse anti-β-actin (catlog A1978) antibody were from Sigma-Aldrich. Rabbit anti-ubiquitin (catlog 3936S), anti-cleaved-IL-1β (catlog 63124S), anti-NLRP3 (catlog 15101S), anti-ASC (catlog 67824S), anti-Gasdermin D (catlog 93709S) and anti-p62 (catlog 39749S) antibodies were from Cell Signaling Technology. Mouse anti-NLRP3 (catlog AG-20B-0014-C100) and anti-Caspase 1 (p20) (catlog AG-20B-0042-C100) antibodies were from AdipoGen. Goat anti-IL-1β antibody

	(catalog AF-401-NA) was from R&D Systems. Rabbit anti-MafB (catlog 20189-1-AP) and mouse anti-GAPDH (60004-1-Ig) were from Proteintech.
Validation	All antibodies are commercially available and were commercially validated.

Eukaryotic cell lines

Policy information about cell lines and Sex and Gender in Research

Cell line source(s)	J774A.1 was from ATCC. Primary mouse macrophages were established as described in the manuscript.
Authentication	Cell line was newly purchased. Primary mouse macrophages were freshly isolated and had expected morphology and characteristics. All culture conditions were well established and extensively described in our previous studies.
Mycoplasma contamination	All cell lines were tested negative for mycoplasma contamination.
Commonly misidentified lines (See ICLAC register)	None

Animals and other research organisms

Policy information about studies involving animals; ARRIVE guidelines recommended for reporting animal research, and Sex and Gender in Research

Laboratory animals	C57BL/6 background wild type and knockout mice were used in this study.
Wild animals	Not applicable
Reporting on sex	Only male mice were used in this study.
Field-collected samples	Not applicable
Ethics oversight	All animal protocols were approved by the University of Alabama at Birmingham Institutional Animal Care and Use Committee (IACUC).

Note that full information on the approval of the study protocol must also be provided in the manuscript.

Flow Cytometry

Plots

Confirm that:

☒ The axis labels state the marker and fluorochrome used (e.g. CD4-FITC).

☒ The axis scales are clearly visible. Include numbers along axes only for bottom left plot of group (a 'group' is an analysis of identical markers).

☒ All plots are contour plots with outliers or pseudocolor plots.

☒ A numerical value for number of cells or percentage (with statistics) is provided.

Methodology

Sample preparation	Sample preparation is described in the manuscript.
Instrument	BD FACSymphony A3
Software	FlowJo v10
Cell population abundance	>10,000 cell events were gated for analysis.
Gating strategy	FSC and SSC parameters were used to gate cell samples and exclude cell debris and large aggregates. Positive and negative populations were defined in the figures of this study.

☒ Tick this box to confirm that a figure exemplifying the gating strategy is provided in the Supplementary Information.
